# Identification of compound heterozygous variants in *MSH4* as a novel genetic cause of diminished ovarian reserve

**DOI:** 10.1186/s12958-023-01127-0

**Published:** 2023-08-24

**Authors:** Yingjing Wan, Zhidan Hong, Binyu Ma, Xuanyi He, Ling Ma, Mei Wang, Yuanzhen Zhang

**Affiliations:** 1 Center for Reproductive Medicine, Zhongnan Hospital of Wuhan University, Wuhan, Hubei P.R. China; 2 Clinical Medicine Research Center of Prenatal Diagnosis and Birth Health in Hubei Province, Wuhan, Hubei P.R. China; 3 Wuhan Clinical Research Center for Reproductive Science and Birth Health, Wuhan, Hubei P.R. China

**Keywords:** *MSH4* variants, Diminished ovarian reserve, Oocyte quality, Large polar body, Bioinformatic analysis, Minigene assay

## Abstract

**Background:**

Diminished ovarian reserve (DOR) is a common cause of female infertility, with genetic factors being a significant contributor. However, due to high genetic heterogeneity, the etiology of DOR in many cases remains unknown. In this study, we analyzed the phenotype of a young woman with primary infertility and performed molecular genetic analysis to identify the genetic cause of her condition, thus providing important insights for genetic counseling and reproductive guidance.

**Methods:**

We collected the patient’s basic information, clinical data, as well as diagnostic and therapeutic history and performed whole-exome sequencing on her peripheral blood. Candidate pathogenic variants were validated by Sanger sequencing in family members, and the pathogenicity of variants was analyzed using ACMG guidelines. We used bioinformatics tools to predict variant effects on splicing and protein function, and performed in vitro experiments including minigene assay and expression analysis to evaluate their functional effects on HEK293T.

**Results:**

We identified biallelic *MSH4* variants, c.2374 A > G (p.Thr792Ala) and c.2222_2225delAAGA (p.Lys741Argfs*2) in the DOR patient. According to ACMG guidelines, the former was classified as likely pathogenic, while the latter was classified as pathogenic. The patient presented with poor oocyte quantity and quality, resulting in unsuccessful in vitro fertilization cycles. Bioinformatics and in vitro functional analysis showed that the c.2374 A > G variant altered the local conformation of the MutS_V domain without decreasing MSH4 protein expression, while the c.2222_2225delAAGA variant led to a reduction in MSH4 protein expression without impacting splicing.

**Conclusions:**

In this study, we present evidence of biallelic variants in *MSH4* as a potential cause of DOR. Our findings indicate a correlation between *MSH4* variants and reduced oocyte quality, as well as abnormal morphology of the first polar body, thereby expanding the phenotypic spectrum associated with *MSH4* variants. Furthermore, Our study emphasizes the importance of utilizing whole-exome sequencing and functional analysis in diagnosing genetic causes, as well as providing effective genetic counseling and reproductive guidance for DOR patients.

**Supplementary Information:**

The online version contains supplementary material available at 10.1186/s12958-023-01127-0.

## Background

Infertility, defined as the inability to conceive after regular, unprotected sexual intercourse for 12 months or longer, affects approximately 48 million couples and 186 million individuals globally, with female factors contributing to 30–50% of the cases [1–4]. Female infertility is a complex condition that can arise from various factors, including endocrine, physiological, anatomical, genetic and immunological disruptions. Advances in sequencing technology have enabled the identification of an increasing number of genetic factors associated with infertility, furthering our understanding of this complex condition and providing important insights into the basic research of reproductive medicine [5]. Diminished ovarian reserve (DOR) and primary ovarian insufficiency (POI) are two inter-related pathologies that contribute to the etiology of female infertility. DOR is characterized by a reduction in the number of ovarian follicles and a decreased responsiveness of the ovaries to ovulation induction drugs. It can be diagnosed based on an abnormal ovarian reserve test (antral follicular count < 5–7 or anti-Mullerian hormone < 0.5–1.1 ng/mL) [6]. POI is a condition in which a woman’s ovaries stop functioning before the age of 40, leading to amenorrhea and infertility, the diagnostic criteria for POI is (i) amenorrhea for at least 4 months; (ii) elevated levels of follicle-stimulating hormone (FSH > 40 IU/l in at least two samples a few weeks apart) and/or low levels of estrogen; (iii) ovarian ultrasound shows no antral follicle [7, 8]. Generally, DOR is considered to be a milder form of ovarian dysfunction and presents with a better prognosis.

The understanding of the etiology of DOR is limited, with current evidence suggesting a complex interplay of genetic and environmental factors. Although variants in several genes have been associated with an increased risk for DOR, including *FMR1*, *FMR2*, *AMHR2*, *LHCGR*, *BMP15*, *TR53*, *GDF9*, *FSHR*, and *NOBOX*, the underlying genetic mechanism remains largely unknown [9, 10]. And the identification of specific genetic causes for DOR is hindered by the complex inheritance pattern and the lack of large-scale genetic studies. Therefore, further investigations are necessary to uncover the genetic basis of DOR and underlying mechanism.

MutS homolog 4 (MSH4) is a protein that belongs to the MutS family, which is responsible for eukaryotic DNA mismatch repair (MMR) [11]. To date, seven members of the MutS family have been identified in eukaryotes, MSH1 to MSH7. Among them, MSH4 and MSH5 are expressed specifically in germ cells and form a heterodimer (MSH4-MSH5) that plays a crucial role in homologous recombination and crossing over during meiosis [12–15]. Other members of the MutS family are widely expressed in various tissues and are crucial for DNA mismatch repair process [16]. Deletion of *msh4* in mice leads to meiotic arrest and increased chromosomal pairing abnormalities, resulting in infertility in both male and female mice [13]. Despite early discoveries of the sterility phenotype in *msh4*^−/−^ mice, the association between *MSH4* gene variants and human infertility was not established until 2017 [13, 17]. Recent studies have linked biallelic variants in *MSH4* to female POI and male NOA, however, the relationship between *MSH4* variants and DOR and their impact on human oocyte quality remains unknown [17–21].

In this study, we have identified biallelic *MSH4* variants in a young DOR patient presenting with poor oocyte quantity and quality. We further investigate the underlying mechanism of the deleterious effect of the two identified variants using bioinformatics tools and in vitro functional analysis.

## Methods

### Case enrollment and ethical approval

A 27-year-old Han woman was admitted to Zhongnan Hospital of Wuhan University with a chief complaint of infertility in December 2020. The study was approved by the Ethics Committee of Zhongnan Hospital of Wuhan University (Approval Code: 2,023,068 K) and an informed written consent was signed by the participant. All procedures involving human participants were performed in accordance with the ethical standards of the Ethics Committee of the Zhongnan Hospital of Wuhan University.

The patient was diagnosed with DOR and underwent two in vitro fertilization (IVF) cycles in our reproductive medicine center. During her first IVF cycle (cycle^#^1), a progestin-primed ovarian stimulation (PPOS) protocol was performed. The protocol was initiated on day 3 of her menstrual cycle with the oral administration of ethinylestradiol (EE) at a dose of 0.035 mg daily and oral cyproterone acetate (CPA) at a dose of 2 mg daily for 4 days. On day 7 of the menstrual cycle, oral medroxyprogesterone acetate (MPA) at a dose of 10 mg daily was added to the treatment regimen. Human menopausal gonadotropin (HMG) was also administered starting on day 3 at a dose of 75IU per day from day 3 to day 6 and 300IU per day from day 7 to day 13.

There was a 5-month interval between the two IVF cycles, during which we implemented artificial cycles as a pre-treatment strategy to optimize antral follicle preparation before initiating the second main IVF cycle. During her second IVF cycle (cycle^#^2), a mild stimulation protocol was performed. The protocol began on day 2 of the menstrual cycle with the initiation of letrozole at a dose of 5 mg per day for 5 days. HMG was also administered starting on day 4 of her menstrual cycle at a dose of 150IU per day from day 4 to day 8 and 225IU per day from day 9 to day 11. On day 11 of the menstrual cycle, 0.25 mg of cetrotide is administered for two days due to LH surge. Ovulation was triggered with human chorionic gonadotropin (HCG) at a dose of 10,000IU when the leading follicle reached 18 mm in size. The oocytes were retrieved and fertilized in the laboratory, and the best-quality embryos were subsequently selected for transfer to the uterus after 5 days of culture.

### DNA isolation

**Fig. 1:**
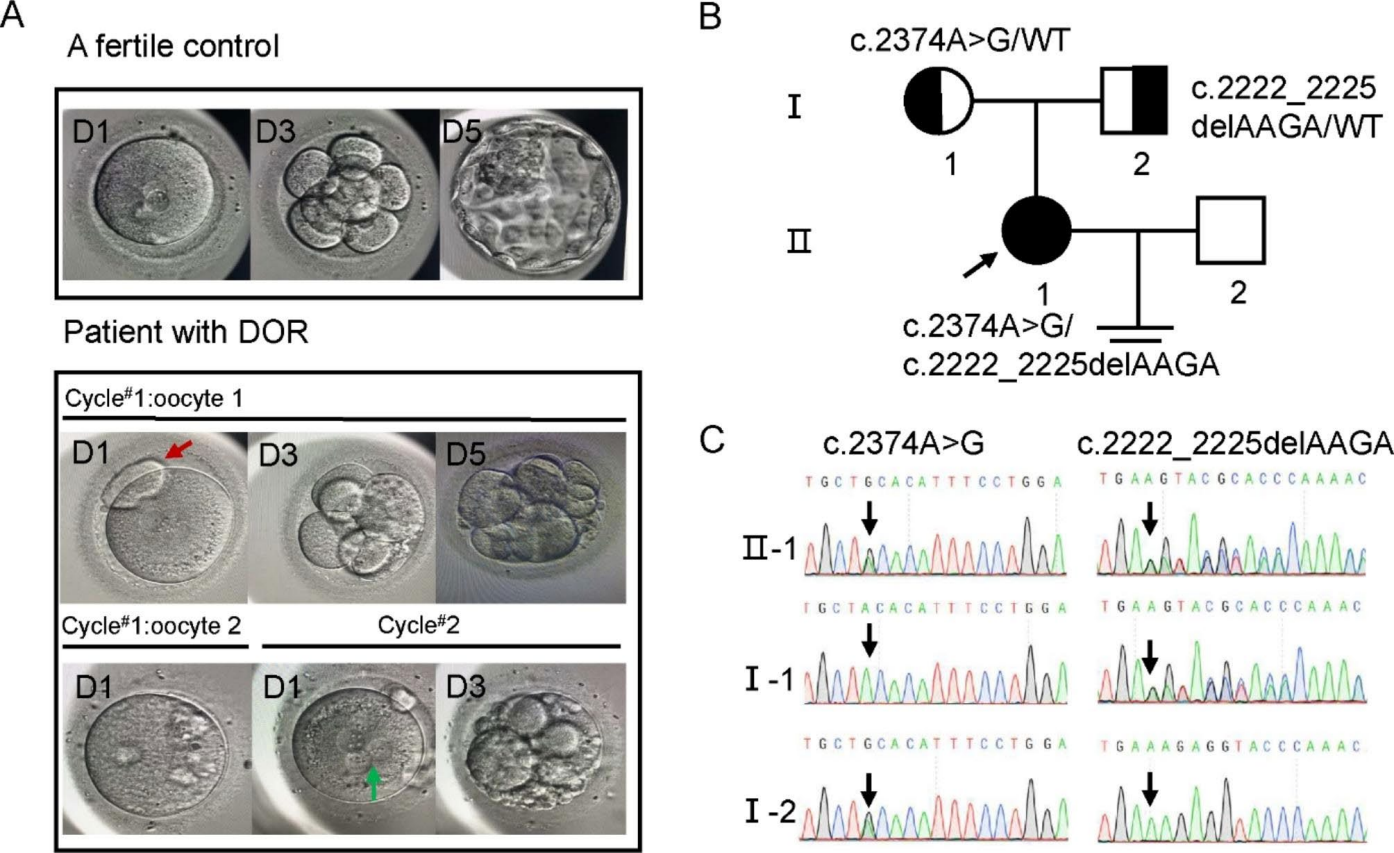
Identification of *MSH4* compound heterozygous variants in a patient with diminished ovarian reserve (DOR) via whole exome sequencing (**A**) The morphology of the oocytes and embryos derived from a fertile control and the DOR patient. In cycle^#^1, two oocytes were obtained, one of which was degenerated and immature, while the other was fertilized but arrested at the 5-day embryonic stage with an enlarged polar body (indicated by a red arrow). In cycle^#^2, only one oocyte was obtained, which was abnormally fertilized and degenerated at the 3-day stage (indicated by a green arrow highlighting the abnormal fertilization with three pronuclei). (**B**) Family pedigree of the patient with DOR, where the proband is indicated by a black circle with an arrow. Semi-filled symbols represent carriers of the heterozygous variants. (**C**) Confirmation of the inheritance pattern of the two variants in *MSH4* gene using Sanger sequencing. The c.2374 A > G variant was inherited from the mother, and the c.2222-2225delAAGA variant was inherited from the father

2mL of peripheral venous blood was collected from the proband (II-1) and their parents (I-1, I-2) with EDTA anticoagulation (Fig. 1C). Whole genome DNA was extracted using a peripheral blood DNA extraction kit (Tiangen Biotech, Beijing, China) according to the manufacturer’s instructions. The obtained DNA samples were then subjected to agarose gel electrophoresis and quantified using a Qubit fluorometer for quality control.

### Whole exome sequencing (WES)

Whole exome sequencing was conducted following BGI (Shenzhen, China) protocols. Genomic DNA was fragmented through sonication and exome capture was performed using the Roche KAPA HyperExome chip. The captured exome regions were then enriched and underwent library construction. Sequencing was performed on the MGISEQ-2000 platform, and sequence reads were mapped to the UCSC hg19 (GRch37) human reference genome using BWA [22]. Variants were called and annotated using a combination of Genome Analysis Toolkit (GATK), ANNOVAR, and custom pipelines. Candidate variants were subsequently validated through Sanger sequencing.

### In silico analysis of variants and prediction of MSH4 protein 3D structure

The potential pathogenicity of the c.2374 A > G variant in *MSH4* gene was predicted by in silico tools including CADD, SIFT, POLYPHEN-2 and Mutation Taster [23–31].The splicing impact of *MSH4* c.2222-2225delAAGA was predicted using SPLICEAI and Human Splicing Finder [31–34].

To study the impact of the *MSH4* variants on the protein’s 3D structure, the homologous model with the highest global QMEANDisCo Score (which reflects the quality of the entire protein structure, with higher values indicating higher quality) was selected from Swiss Model database (Structure ID: O15457) [35]. And structures of the mutant MSH4 protein were generated using site-directed mutagenesis in Pymol v2.6.0 [36].

### Minigene assay

**Fig. 2:**
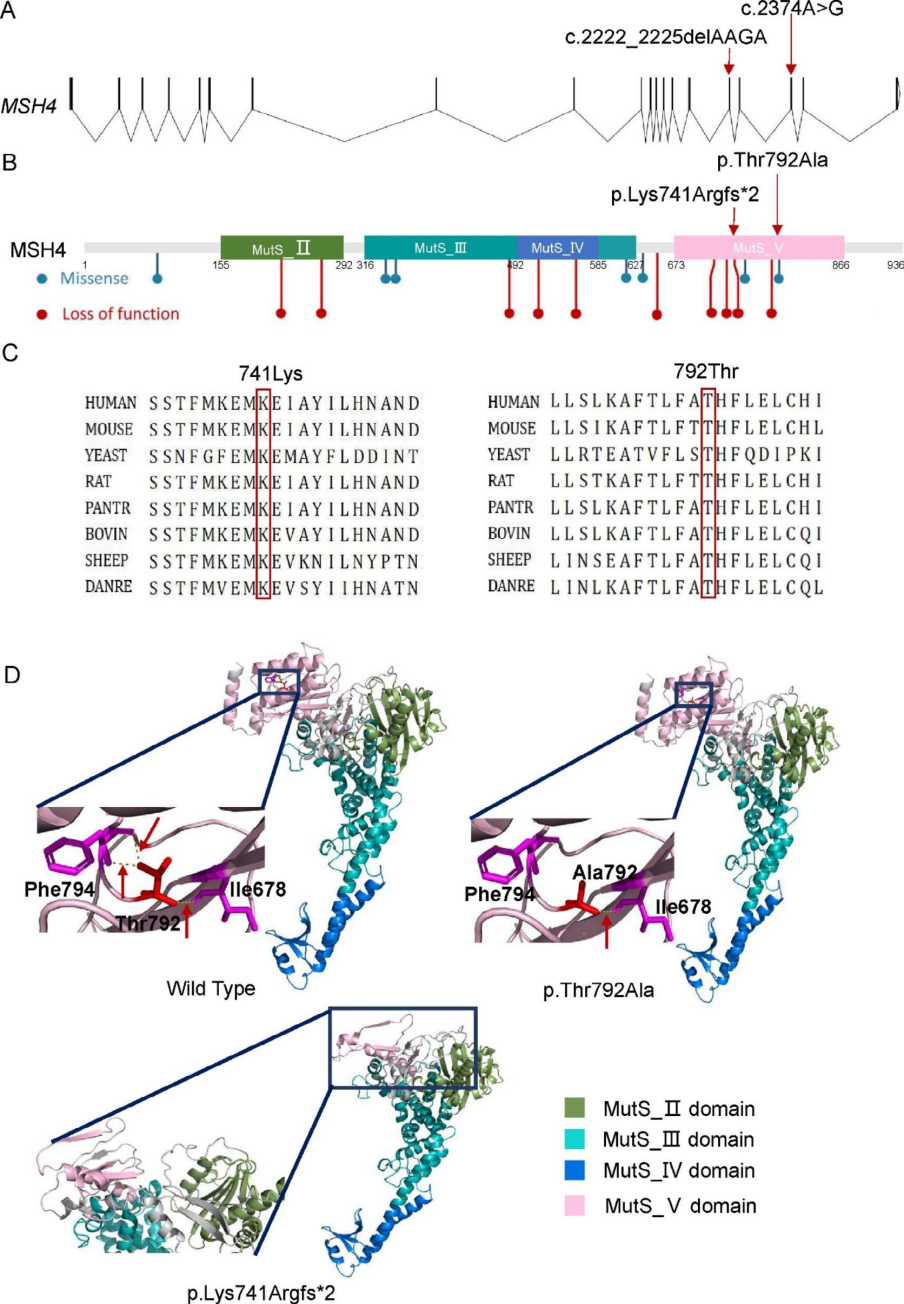
Minigene Analysis indicates *MSH4* c.2222_2225delAAGA does not change splicing. (**A**, **E**) Schematic illustration of minigene construction. To construct the pcDNA3.1-MSH4-wt/mut minigene, exon 15 (201 bp), a portion of intron 15 (469 bp), exon 16 (119 bp), intron 16 (1326 bp), and exon 17 (129 bp) of the *MSH4* gene were cloned into the pcDNA3.1 vector. To construct the pcMINI-MSH4-wt/mut minigene, a portion of intron 15 (425 bp), exon 16 (119 bp) and a portion of intron 16 (576 bp) of *MSH4* gene was cloned into the pcMINI vector. (**B**, **F**) Sanger sequencing results of the minigene constructs. (**C**, **G**) RT-PCR products of the minigenes expressed in HeLa and 293T cells. (**D**, **H**) Sanger sequencing of the amplified products revealed no significant difference in splicing between the wild-type and mutant minigenes

The variant *MSH4* c.2222_2225delAAGA was located at 3’ boundary of exon 16 of *MSH4* gene. To investigate its splicing effect, we constructed wild-type (wt) and mutant (mut) minigenes based on two different vector systems and the sequence were subsequently verified by Sanger sequencing. In the pcDNA3.1-MSH4-wt/mut minigene, exon 15 (201 bp), a segment of intron 15 (469 bp), exon 16 (119 bp), intron 16 (1326 bp) and exon 17 (129 bp) of *MSH4* gene were cloned into the pcDNA3.1 vector (Bioeagle, Wuhan, China) (Fig. 2A). In the pcMINI-MSH4-wt/mut minigene, a segment of intron 15 (425 bp), exon 16 (119 bp) and a segment of intron 16 (576 bp) of *MSH4* gene were cloned into the pcMINI vector (Bioeagle, Wuhan, China) (Fig. 2E). Constructs were then transfected into HEK293T and HeLa cells using Lipofectamine 2000 (Invitrogen, America) following the manufacturer’s protocol. The cells were collected 48 h after being transfected and total RNA was extracted using Trizol (TaKaRa, Otsu, Japan) according to the manufacturer’s protocol. Reverse transcription was then performed using the Hifair® II 1st Strand cDNA Synthesis SuperMix for qPCR (gDNA digester plus) kit (Yeasen, Shanghai, China) according to the manufacturer’s instructions. The cDNA was amplified by PCR, and the PCR products were analyzed by electrophoresis on a 1.5% agarose gel and Sanger sequencing. The primers used are listed in Supplementary Table 1.

### Construction of *MSH4* expression plasmid

The wild-type Flag-*MSH4* expression plasmid was generated by fusing the full-length wild-type *MSH4* cDNA fragment into pcDNA3.1 plasmid (Bioeagle, Wuhan, China) with an N-terminal Flag tag. Variants were introduced into the wild-type cDNA using site-directed mutagenesis. Then the mutant *MSH4* cDNA was cloned into pcDNA3.1 plasmid (Bioeagle, Wuhan, China) with an N-terminal Flag tag to construct mutant Flag-*MSH4* expression plasmid. All plasmids were confirmed by Sanger sequencing.

### Cell culture and transfection

HEK293T and HeLa cells were cultured in Dulbecco’s Modified Eagle Medium (DMEM, Gibco, Thermo Fisher Scientific, USA) with 10% FBS and 1% penicillin-streptomycin at 37 °C and 5% CO2. Cells were passaged every 3–4 days using trypsinization and subcultured at ratios of 1:3 to 1:5. Cells were plated 24 h before transfection. HeLa or HEK293 cells were transiently transfected with plasmid using Lipofectamine 2000 reagent(Invitrogen, America)and OPTI-MEM (Gibco, Thermo Fisher Scientific, USA) according to manufacturer’s protocols. Transfected cells were incubated for 48 h to allow transient protein expression.

### Quantitative polymerase chain reaction (qPCR)

After 48 h of transfection with wild-type and mutant expression plasmid, cell samples were collected. Total RNA was extracted using Trizol (TaKaRa, Japan) and cDNA was synthesized using the Hifair® II 1st Strand cDNA Synthesis SuperMix for qPCR (gDNA digester plus) kit (Yeasen, Shanghai, China) according to the manufacturer’s protocol. Quantification of gene expression was performed using qPCR with the ABI Prism 7500 (Applied Biosystems). The cDNA was used as template in qPCR reactions with specific primers and probes for *MSH4*, as well as *GAPDH* as control. Thermal cycling conditions consisted of an initial denaturation at 95 °C for 10 min, followed by 40 cycles of 95 °C for 15 s and 60 °C for 1 min. Data were analyzed using the 2^(-ΔCt) method, and significance was determined using a t-test with a significance level of p < 0.05. The primers used are listed in Supplementary Table 1.

### Western blot

Protein expression was analyzed by Western blot. Cells were lysed in 1% Triton X-100, 1 mM EDTA, 0.1% SDS, 1 mM PMSF buffer. Proteins were separated by 10% SDS-PAGE and transferred to nitrocellulose membranes. Membranes were blocked with 5% non-fat dry milk in TBST for 1 h at room temperature. Membranes were incubated with primary antibody anti-Flag (1:2000 dilution, DIAAN, Wuhan, China) overnight at 4 °C, washed and incubated with horseradish peroxidase-conjugated secondary antibody (1:5000 dilution, Thermo Fisher Scientific, USA) for 1 h at room temperature. Proteins were detected using ECL substrate (Thermo Scientific, USA) and visualized on X-ray film. Densitometry was performed using Image J, data presented as ratio of target protein to internal control. The statistical significance of the data was determined using a t-test with a significance level of p < 0.05.

## Results

### Patient’s clinical manifestations

The proband II-1 from a non-consanguineous Han Chinese family was diagnosed with DOR at the age of 27 (Fig. 1B). She came to our reproductive medicine center with a chief complaint of primary infertility. She got married 3 years ago and failed to get pregnant since then. Despite undergoing multiple follicle monitoring cycles at other hospitals, she had not yet been able to conceive. She experienced menarche at the age of 13 and had regular menstrual cycles every four weeks, lasting 5–6 days. She had no history of sexually transmitted disease, pelvic inflammatory disease, use of an intrauterine device or exposure to diethylstilbestrol. Physical examinations indicated normal stature, BMI and normal development of the genitalia, hair distribution as well as normal secondary sexual characteristics. Karyotyping results complied with apparent normal females (46, XX), and the copy number of *FMR1* CGG repeats was within the normal polymorphic range. The basic hormone levels of the proband were as follows: follicle-stimulating hormone (FSH), 29.86 mIU/mL; luteinizing hormone (LH), 4.67 mIU/mL; estradiol (E2), 2.29 pg/mL; testosterone (T), 0.3 ng/mL; prolactin (PRL), 12.98 ng/mL and anti-Mullerian hormone (AMH), 0.54 ng/mL. Transvaginal ultrasound showed normal-sized uterus and ovaries with two antral follicles in the left ovary and one in the right ovary. These finding strongly supported that this patient was a case of idiopathic primary DOR (summarized in Table 1).

**Table 1:** Clinical characteristics of the DOR patient

Clinical features	
Age(y)	27
History of infertility(y)	3
Menstrual cycle	normal
Physical examination	
BMI(kg/m^2^)	22.59
External genitalia	normal
Secondary traits	normal
Antral follicle count (AFC)	3*
Somatic karyotype	46, XX
FMR1 CGG repeats	normal
Sex hormone levels	
Follicle-stimulating hormone (mIU/mL)	29.86*
Luteinizing hormone (mIU/mL)	4.67
Testosterone (ng/mL)	0.3
Estradiol (pg/mL)	2.29
Prolactin (ng/mL)	12.98
Anti-mullerian hormone(ng/mL)	0.54*

This table summarizes the clinical characteristics of a patient with diminished ovarian reserve (DOR). The patient‘s age, history of infertility, menstrual cycle, physical examination results, antral follicle count, somatic karyotype, and sex hormone levels are included. *indicates outside the normal range. Normal range of AFC: 7–20; Follicle-stimulating hormone: 3–12 mIU/mL; Anti-mullerian hormone: 1.39-6.42ng/mL

The DOR patient underwent two IVF cycles in our reproductive medicine center, with specific details of the ovarian stimulation protocols provided in the Methods section of the manuscript. In cycle^#^1, two oocytes were obtained, one oocyte degenerated and remained immature, while another, displaying an enlarged polar body, was fertilized but arrested in embryonic development (Fig. 1A). In cycle^#^2, only one oocyte was obtained, but it was abnormally fertilized, displaying three pronuclei (3PN) and subsequently degenerated (Fig. 1A).

### WES identified compound heterozygous variants in *MSH4* in the DOR patient

Considering the patient had unexplained diminished ovarian reserve, and the quality of retrieved oocytes during her two IVF cycles were poor, a genetic etiology cannot be ruled out. To investigate the underlying genetic basis, WES was performed on the patient, generating 23,550 Mb of raw data.

The targeted region covered a length of 42,836,424 bp with 100% coverage and an average depth of 327.07X. Our analysis showed that 99.92% of the targeted regions had a depth of 10X or more, while 99.81% had a depth of 20X or more. Through WES, we identified compound heterozygous *MSH4* variants, c.2374 A > G (p.Thr792Ala) and c.2222_2225delAAGA (p.Lys741Argfs*2) in the DOR patient, Sanger sequencing indicated that the c. 2374 A > G variant was inherited from her mother, while the c.2222_2225delAAGA variant was inherited from her father (Fig. 1C).

### In silico prediction of variants’ impact on MSH4 protein function

**Fig. 3:**
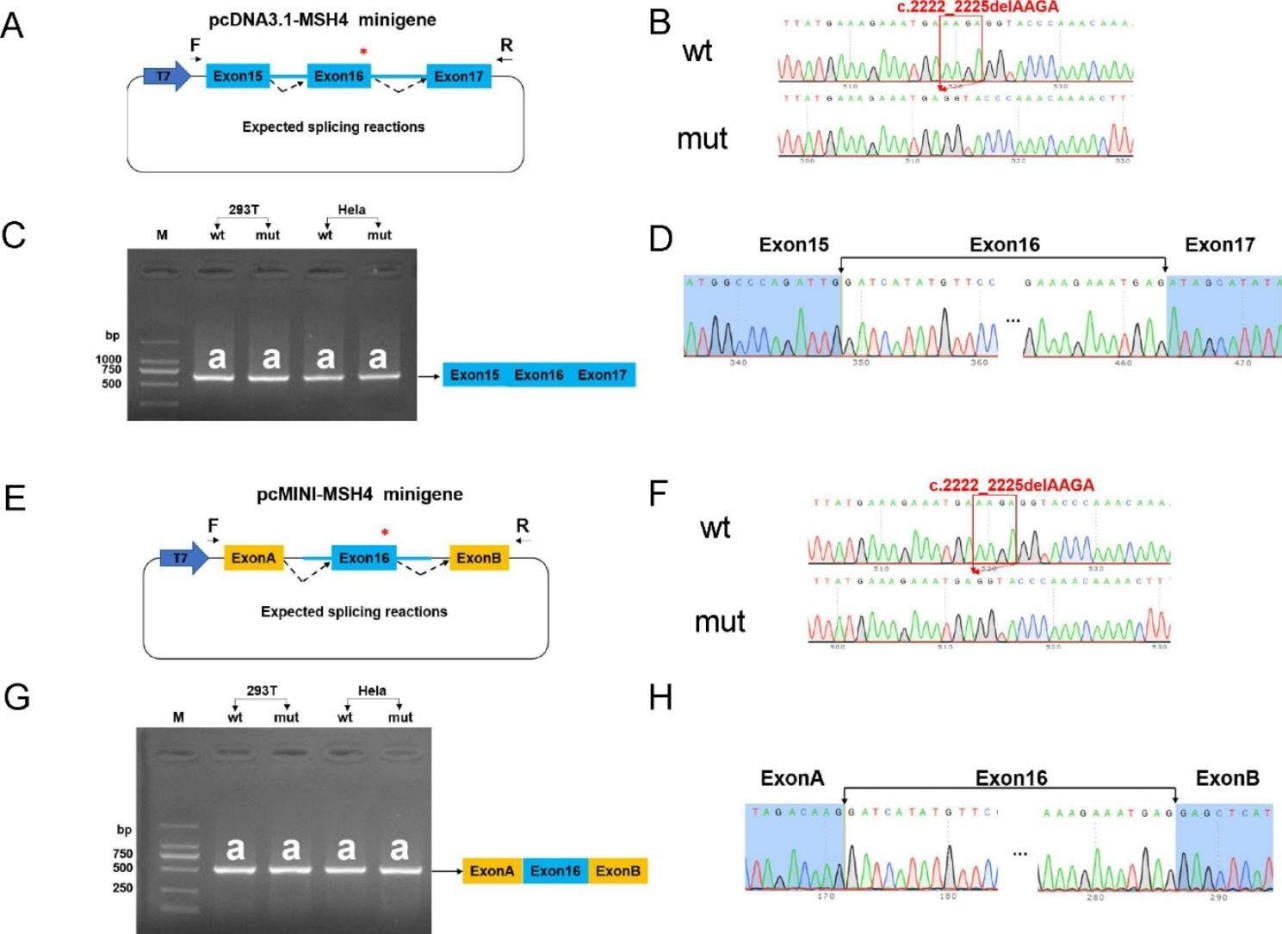
In silico evaluation indicates *MSH4* variants potentially influence protein function (**A**) A schematic diagram illustrating the exon-intron structure of the *MSH4* gene, with the genomic locations of the c.2374 A > G (located in exon 18) and c.2222-2225delAAGA (located in exon 16) variants indicated. (**B**) A schematic representation of the MSH4 protein domains, including the locations of disease-associated genetic variants from Clinvar database and literature. Blue dots indicates missense disease-causing variants, and red dots represents loss of function disease-causing variants. (**C**) Multiple sequence alignments of MSH4 proteins from various species, demonstrating the high conservation of residues 741 (Lys) and 792 (Thr). (**D**) 3D structural prediction of MSH4 after site-directed mutagenesis using Pymol v2.6.0. At 792 position, three hydrogen bonds(H-bond) are observed with Ile 678 and Phe794 in wild type(Thr792), and two of them are abolished due to the replacement of Ala792. And p.Lys741Argfs*2 results in the loss of a substantial portion of the MutS_V domain

The missense variant c.2374 A > G (rs557796016) was rare, with an allele frequency of 0.00003527, had not been reported in a homozygous state according to the gnomAD database. And it was classified as likely pathogenic according to the ACMG guidelines, with a PM2 + PM3 + PP3 + PP4 rating in this DOR patient. This variant was located in exon 18 of the *MSH4* gene (Fig. 3A), led to the substitution of a threonine residue with alanine in the MutS_V domain (Fig. 3B). Threonine and alanine were both neutral amino acids, however, threonine had a polar, hydrophilic side chain, while alanine had a non-polar, hydrophobic aliphatic side chain. Multiple sequence alignments revealed high conservation of this residue among homologs (Fig. 3C). In silico analysis using various variant effect prediction tools, including CADD, SIFT, POLYPHEN-2 and MutationTaster, suggested that this variant was deleterious [23–26].

To further investigate the impact of the variant c.2374 A > G (p.Thr792Ala) on the 3D structure of the MSH4 protein, we selected the homologous model with the highest global QMEANDisCo Score from Swiss Model database, and constructed the mutant MSH4 using site-directed mutagenesis with Pymol v2.6.0 (Fig. 3D) [35, 36]. Our 3D structure prediction showed that the Thr792 residue, located in the MutS_V domain of MSH4, formed two hydrogen bonds with Phe794 and one hydrogen bond with Ile678. The substitution of Thr with Ala resulted in a shorter side chain and the loss of the hydrogen bond between Thr and Phe794, leading to a local conformational change in the protein that may affect its stability and function (Fig. 3D).

The *MSH4* c. 2222_2225delAAGA (rs1386320504) variant was rare, with an allele frequency of 0.000004692 and had not been reported in a homozygous state according to gnomAD database. And it was classified as pathogenic according to the ACMG guidelines, with a PVS1 + PM2 + PP4 rating in this DOR patient. This variant was located at the boundary of exon 16 of the *MSH4* gene, just two base pairs upstream of the canonical donor site. To assess the potential impact of this variant on splicing, we employed bioinformatic tools SPLICEAI and Human splicing finder (HSF). SPLICEAI predicted a high Δ score of 0.91, indicating a strong possibility of alteration in splicing due to donor loss [33]. HSF predicted that this variant could result in donor loss, activation of a cryptic donor site, and significant changes in exon splicing enhancer/exon splicing silencer (ESE/ESS), potentially leading to splicing alteration [37].

Although splicing prediction bioinformatic tools were initially used to analyze the c.2222_2225delAAGA (p.Lys741Argfs*2) variant, given their potential limitations, we performed additional analyses to evaluate the impact of this variant on the structure and stability of the MSH4 protein, independent of any potential splicing alterations. Multiple sequence alignments of this amino acid showed a high degree of conservation among homologs (Fig. 3C). Additionally, structural predictions suggested that this variant led to the loss of a substantial portion of the MutS_V domain, which may result in alterations to the protein’s structure and function (Fig. 3D).

**Table 2:** An overview of *MSH4* disease-causing variants from Clinvar database and literature

Nucleotide change	Protein change	Type of mutation	Sex	Associated Phenotype	Clinvar ID	Reference
c.G244A	p.Gly82Ser	missense	male	NOA	—	Li P et al., 2022 [18].
c.670delT	p.Leu224Cysfs*3	frameshift	male	NOA	—	Li P et al., 2022 [18].
c.805_812del	p.Val269Glnfs*15	frameshift	male	NOA	1,693,502	Li P et al., 2022 [18].
c.1025 C > T	p.Thr342Ile	missense	female	POI	1,256,043	—
c.1063 A > G	p.Ile355Val	missense	female	POI	1,255,997	—
c.1453 C > T	p.Gln485Ter	nonsense	male	NOA	992,887	Wyrwoll MJ et al., 2021 [19].
c.1552 C > T	p.Gln518Ter	nonsense	male	NOA	1,693,500	Tang D et al., 2020 [38].
c.1686del	p.Lys562_Val563insTer	nonsense	male	NOA	992,888	Wyrwoll MJ et al., 2021 [19].
c.1855 A > G	p.Met619Val	missense	female	POI	1,256,012	—
c.1913 C > T	p.Pro638Leu	missense	male	NOA	—	Krausz et al., 2020 [21]
c.1950G > A	p.Trp650Ter	nonsense	male	NOA	1,693,503	Li P et al., 2022 [18].
c.2179del	p.Asp727Metfs*11	frameshift	male	NOA	1,693,504	Li P et al., 2022 [18].
c.2198 C > A	p.Ser733Ter	nonsense	male/female	NOA,POI	992,889	Wyrwoll MJ et al., 2021 [19].
c.2222_2225del	p.Lys741Argfs*2	frameshift	male/female	NOA, POI	1,256,044	Li P et al., 2022 [18].
c.2261 C > T	p.Ser754Leu	missense	male/female	NOA,POI	869,115	Krausz et al., 2020 [21]; Akbari A et al., 2021 [20].
c.2355 + 1G > A	——	splice site	female	POI	1,693,499	Carlosama C et al., 2017 [17].
c.2374 A > G	p.Thr792Ala	missense	female	POI	1,256,001	—
c.2728 C > T	p.Arg910Ter	nonsense	female	POI	1,256,045	—

The table summarized previously reported *MSH4* disease-causing variants from ClinVar database and literature, including nucleotide and protein changes, mutation types, sex of the patients, associated phenotypes, ClinVar IDs, and references

### Analysis of *MSH4* c. 2222_2225delAAGA’s impact on splicing using minigene assay

To assess the effect of *MSH4* c.2222_2225delAAGA on splicing, we generated wild-type and mutant *MSH4* minigenes using two distinct vector systems (Fig. 2A, E) and confirmed their accuracy and integrity through Sanger sequencing (Fig. 2B, F). The minigenes were then transfected into HEK 293T and Hela cells, and splicing patterns were analyzed by isolating mRNA and conducting RT-PCR and Sanger sequencing. RT-PCR analysis from both vector systems revealed that the wild-type and mutant minigenes produced similar spliced products, indicating that the c.2222_2225delAAGA variant did not result in abnormal splicing (Fig. 2C, G). The Sanger sequencing results were in agreement with the RT-PCR analysis, providing additional evidence that the *MSH4* c.2222_2225delAAGA variant did not affect splicing in vitro (Fig. 2D, H).

### In vitro expression analysis of *MSH4* variants, c.2374 A > G and c.2222_2225delAAGA

**Fig. 4:**
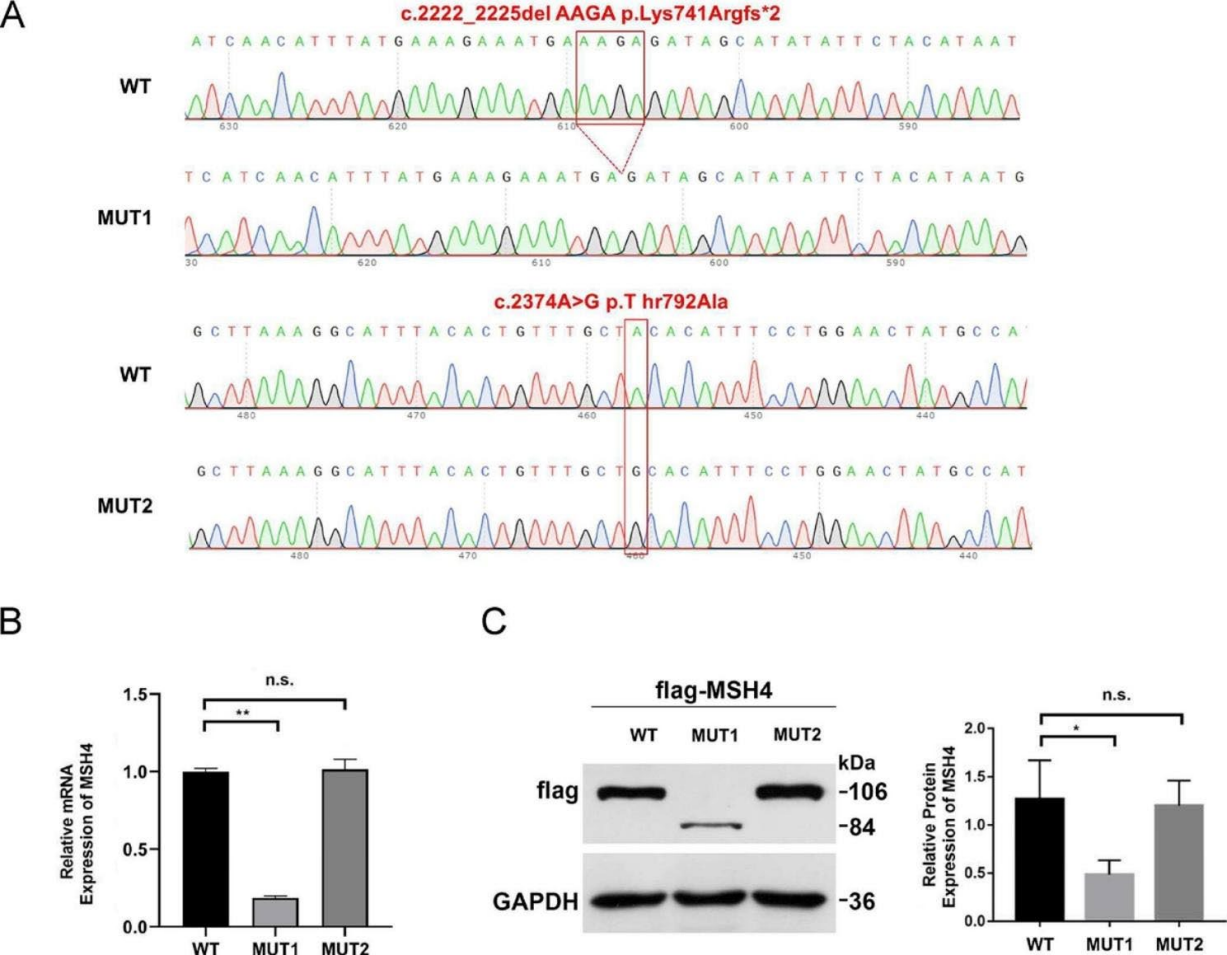
In vitro expression experiments in HEK293T cells show decreased MSH4 expression with c.2222-2225delAAGA (MUT1) variant, but no change with c.2347 A > G (MUT2) variant. (**A**) Sanger sequencing confirmed the successful construction of MSH4 expression plasmids. (**B**) RT-qPCR analysis showed *MSH4* mRNA downregulation in total RNA extracted from HEK293T transfected with MUT1 expression plasmids. Results were normalized against GAPDH. Data was presented as mean ± SEM. **P = 0.01. (**C**) Western blot analysis of protein extracts from HEK293 cells transfected with WT, MUT1 and MUT2 expression plasmids. MSH4 was tagged with a Flag epitope and detected with a Flag antibody, while GAPDH served as a loading control. Data was presented as mean ± SEM. *P = 0.05

To evaluate the impact of the c.2374 A > G and c.2222_2225delAAGA variants on *MSH4* expression, HEK 293T cells were transfected with both wild-type and mutant *MSH4* expression plasmids. mRNA and protein levels were quantified through quantitative PCR and western blot analysis, respectively. Our results showed that the *MSH4* c.2222_2225delAAGA variant had a significant decrease in both mRNA (p < 0.05) and protein expression compared to the wild-type (Fig. 4B, C). On the other hand, no significant alterations were detected in MSH4 expression levels for the c.2374 A > G variant (Fig. 4B,C). These findings suggest that the c.2222_2225delAAGA variant resulted in a reduction of MSH4 protein expression, while the c.2374 A > G variant had no significant effect on MSH4 expression in vitro.

## Discussion

In this study, we report on the oocyte phenotype and IVF outcomes in a female with biallelic deleterious variants of the *MSH4* gene. During two IVF cycles, three oocytes were retrieved, but none resulted in viable embryos, suggesting a possible defect in oocyte maturation and fertilization. During the first IVF cycle, an oversized first polar body was observed. Previous studies have linked variants in genes like *TUBB8*, *PATL2*, *TRIP13*, *TBPL2*, *PANX1*, *MOS*, *ZP1*, *ZP2*, and *ZP3*, to the formation of an enlarged polar body [39]. Our findings are in agreement with previous studies, which have proposed a link between the presence of large polar bodies and poor oocyte quality as well as inferior outcomes in IVF [40]. For the first time, we suggest that *MSH4* variants may impact not only oocyte quantity but also oocyte quality and result in abnormal polar body morphology. However, further investigation into the underlying mechanisms is not feasible due to ethical limitations and the unavailability of the patient’s ovarian samples.

MSH4, a member of the MutS family, forms a complex with MSH5. Unlike other members of the MutS family involved in DNA mismatch repair, MSH4 and MSH5 are specificially expressed in germ cells and play a critical role in meiotic recombination. The MSH4-MSH5 heterodimer acts as a sliding clamp that stabilizes the intermediate structure during DNA strand exchange. It encompasses the DNA, holding the two strands together, and facilitates the action of recombination proteins [12, 41]. MSH4 and MSH5 are both composed of four domains: MutS_II, MutS_III, MutS_IV, and MutS_V (Fig. 3B). The MutS_II domain (connector domain) connects the MutS_III and MutS_V domains (Fig. 3D), while the MutS_III domain (core domain) consists of two subdomains that form a helical bundle and act as levers extending towards the DNA. The MutS_IV domain (clamp domain) is located between the two subdomains of the Mut_III domain at the top of the lever helices, and the MutS_V domain (ATPase domain) features a classical Walker A motif [42]. Deletion of *msh4* in mice leads to meiotic arrest at the diplotene stage and increased chromosomal pairing abnormalities, resulting in infertility in both male and female mice [13].

Despite the fact that sterility phenotype in *msh4*^−/−^ mice was discovered as early as 2000, the association of *MSH4* gene variants with human infertility was not established until 2017 [13, 17]. In 2017, Carlosama et al. reported the first case of the association between *MSH4* variants and POI in a family with a homozygous splice site variant, *MSH4* c.2355 + 1G > A. And in vitro minigene assay suggested that it led to exon 17 skipping [17]. In 2020, Tang et al. reported the first case of NOA caused by a homozygous nonsense variation, *MSH4* c.1552 C > T (p.Q518X) in a consanguineous family. Testis biopsy as well as hematoxylin and eosin staining of testicular tissue suggested meiotic arrest at diplotene stage [38]. Since then, several studies have further confirmed the association of *MSH4* variants with both NOA and POI (summarized in Table 2; Fig. 3B) [18–21]. In this study, we present the case of a young infertile female who carries compound heterozygous *MSH4* variants, 2374 A > G (p.Thr792Ala) and c.2222_2225delAAGA (p.Lys741Argfs*2). Unlike previously reported cases of POI, the patient has a milder phenotype and is diagnosed with DOR. She has undergone two IVF cycles in our center and obtained three oocytes, however, no viable embryos were available for transplantation (Table 1; Fig. 1). In this study, we identify biallelic *MSH4* variants as a novel cause of DOR, expanding the phenotypic spectrum of *MSH4* variants and highlighting the importance of early genetic screening for DOR patients with unknown etiology.

The *MSH4* c.2374 A > G variant (p.Thr792Ala) has been recorded in the Clinvar database, but its potential impact has not been documented in the literature. This study utilized bioinformatics tools to predict the impact of the variant and analyzed the 3D structures of both the wild-type and mutant MSH4 proteins. The variant was located within the evolutionarily conserved MutS_V domain, which plays a crucial role in DNA repair across different species. Our findings indicate that this variant disrupts hydrogen bond formation and alters the local spatial conformation within this domain. In vitro experiments using HEK 293T cells showed that the variant does not affect MSH4 protein expression. In conclusion, the *MSH4* c.2374 A > G variant likely exerts its effects by altering the local conformation of the MutS_V domain rather than through haploinsufficiency. However, further studies are needed to fully understand the impact of the variant on the spatial structure of MSH4, its interaction with MSH5, and the binding of the MSH4-MSH5 complex to Holliday junctions.

The *MSH4* c.2222_2225delAAGA variant has been previously associated with NOA and meiotic arrest, but its impact has not been validated through in vitro experiments [18]. Considering the variant’s proximity to the canonical donor site and the positive results of splicing bioinformatic prediction tools, we transfected *MSH4* minigenes into HEK293T cells. Our results indicated that the variant does not alter the splicing pattern. Under the canonical splicing pattern, the c.2222_2225delAAGA variant leads to a premature termination codon (PTC), which is likely to decrease protein expression through nonsense-mediated mRNA decay (NMD) pathway [43, 44]. To confirm this effect, we compared the mRNA and protein levels of the wild-type and mutant *MSH4* using expression plasmids transfected into 293T cells. Our experiments show that the variant results in a significant decrease in mRNA and protein levels. This study highlights the limitations of bioinformatics tools and emphasizes the importance of experimental validation as the gold standard for determining the impact of genetic variants.

## Conclusions

In this study, we have provided new evidence supporting the association between *MSH4* biallelic variants and diminished ovarian reserve (DOR). Our findings suggest that these variants may affect not only oocyte quantity but also its quality. Furthermore, we have validated our bioinformatics predictions through in vitro experiments and demonstrated that the *MSH4* c.2374 A > G (p.Thr792Ala) variant affects protein function through alterations in local spatial conformation, rather than haploinsufficiency, while the *MSH4* c.2222_2225delAAGA variant leads to haploinsufficiency by reducing protein expression, rather than by affecting splicing. These results contribute to a better understanding of the underlying genetic mechanisms of DOR and have potential implications for the development of new diagnostic and therapeutic approaches for this condition.

### Electronic supplementary material

Below is the link to the electronic supplementary material.

Supplementary Material 1

## Data Availability

All data generated or analyzed during this study are included in this published article and its supplementary information files.

## Abbreviations

DOR: Diminished ovarian reserve
POI: Primary ovarian insufficiency
NOA: Non-obstructive azoospermia
MSH4: MutS homolog 4
MMR: Mismatch repair
GATK: Genome Analysis Toolkit
WES: Whole exome sequencing
qPCR: Quantitative polymerase chain reaction
IVF: In vitro fertilization
FSH: Follicle-stimulating hormone
LH: Luteinizing hormone
E2: Estradiol
T: Testosterone
PRL: Prolactin
AMH: Anti-Mullerian hormone
PPOS: Progestin-primed ovarian stimulation
EE: Ethinylestradiol
CPA: Cyproterone acetate
MPA: Medroxyprogesterone acetate
HMG: Human menopausal gonadotropin
HCG: Human chorionic gonadotropin
PTC: Premature termination codon
NMD: Nonsense-mediated mRNA decay
